# Photosymbiotic ascidians from Pari Island (Thousand Islands, Indonesia)

**DOI:** 10.3897/zookeys.422.7431

**Published:** 2014-06-30

**Authors:** Euichi Hirose, Budhi Hascaryo Iskandar, Yusli Wardiatno

**Affiliations:** 1 Department of Chemistry, Biology and Marine Science, Faculty of Science, University of the Ryukyus, Nishihara, Okinawa 903-0213, Japan; 2 Department of Fishery Resources Utilization, Faculty of Fisheries and Marine Science, Bogor Agricultural University, Gedung FPIK Lt. 3, Kampus IPB Darmaga, Bogor 16680, Indonesia; 3 Department of Aquatic Resources Management, Faculty of Fisheries and Marine Science, Bogor Agricultural University, Gedung FPIK Lt. 3, Kampus IPB Darmaga, Bogor 16680, Indonesia

**Keywords:** Algal symbiosis, Colonial ascidian, Biogeography, Coral reefs, Didemnidae

## Abstract

Photosymbiotic ascidian fauna were surveyed in the subtidal zone off Pari Island in the Thousand Islands (Java Sea, Indonesia). Nine species were recorded: *Didemnum molle*, *Trididemnum miniatum*, *Lissoclinum patella*, *L. punctatum*, *L. timorense*, *Diplosoma gumavirens*, *D. simile*, *D. simileguwa*, and *D. virens*. All of these species have been previously recorded in the Ryukyu Archipelago, Japan. *Diplosoma gumavirens* and *D. simileguwa* were originally described from the Ryukyu Archipelago in 2009 and 2005, respectively, and all of the observed species are potentially widely distributed in Indo–West Pacific coral reefs.

## Introduction

In tropical waters, some colonial ascidians harbor cyanobacterial symbionts such as *Prochloron* (reviewed by Lewin and Cheng 1989; E. Hirose et al. 2009a; Hirose 2014). The host ascidians always belong to the family Didemnidae, which is likely the largest family in the class Ascidiacea (e.g., Kott 2004; Shenkar et al. 2011). Although photosymbiotic didemnids are sometimes more common than any other ascidians in shallow coral reefs, they are often overlooked because of their small size and cryptic habitats. In contrast, they have been attractive sources of bioactive compounds for researchers in the biochemical and pharmaceutical sciences (e.g., Schmidt et al. 2012). To date, about 30 species in the four didemnid genera (*Didemnum*, *Diplosoma*, *Lissoclinum*, and *Trididemnum*) have been recorded as host species worldwide (e.g., Kott 2001). However, the ranges of distribution of individual species are less understood, as few faunal records of photosymbiotic ascidians exist (e.g., Kott 2001; Monniot and Monniot 2001). To identify these species, it is often necessary to examine zooid morphology under a microscope. Therefore, reexamination may be necessary for some records in older publications.

The Pulau Pari Technical Management Unit for Human Resources Development on Oceanography Competency is a marine laboratory located on Pari Island (Thousand Islands, Indonesia). This laboratory of the Indonesian Institute of Sciences (LIPI) is one of the key stations for marine science in the Java Sea. Therefore, acquiring biodiversity data in this area is essential. Here, we report the photosymbiotic ascidian fauna observed in the shallow coral reef in the vicinity of this laboratory.

## Materials and methods

**Figure 1.**
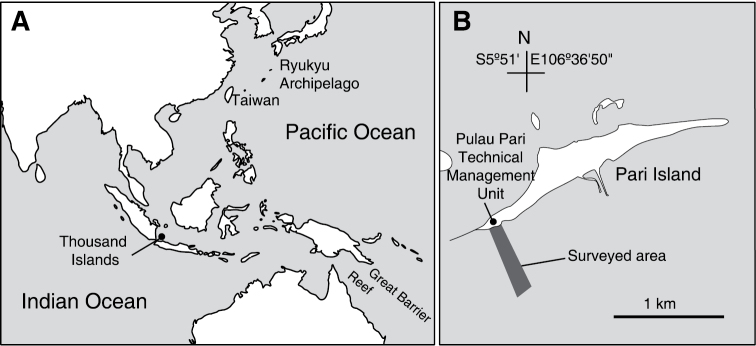
Location of Thousand Islands, Indonesia (**A**) and the surveyed area off Pari Island (**B**).

Samples were collected by snorkeling in the shallow subtidal zone down to approximately 2 m or less at low tide in the back reef, reef flat, and reef crest off Pari Island (5°52'S, 106°36'40"E) on 28–30 November 2013 (Fig. 1). Ascidian colonies were photographed *in situ* prior to collection. Specimens were anesthetized using menthol and 0.37 M MgCl_2_ for approximately 2 h and then fixed with 10% formalin–seawater. Fixed colonies were dissected under a binocular stereomicroscope. Zooids and spicules were photographed using a microscope equipped with differential interference contrast optics. In some photomicrographs of the thoraxes, several images were combined to increase the depth of field using the post-processing image software Helicon Focus Pro 4.2.8 (Helicon Soft, Ltd., Kharkov, Ukraine). Cyanobacterial symbionts were identified based on the colour in live specimens and the cytomorphology under a light microscope. Ascidian taxa were mainly identified following Kott (2001) and Hirose and Su (2011). The work by Shenkar et al. (2014) was also consulted for synonyms. Specimens were deposited in the Museum Zoologicum Bogoriense, Research Institute for Biology, Indonesian Institute of Science (LIPI), Indonesia.

## Results

Nine photosymbiotic ascidian species were found in the subtidal zone of the coral reef off Pari Island. Symbiont cyanobacteria within all ascidian species were identified as *Prochloron didemni* that is the only taxonomically valid species. Depending on the host species, *Prochloron* cells were distributed in the common cloacal cavities, in the tunic, or in both the common cloacal cavity and the tunic. Although the Prochloron cells in the cavity are morphologically different from those in the tunic (Cox 1986), they are indistinguishable genetically (Münchhoff et al. 2007; Hirose et al. 2012).

### Didemnum molle

Taxon classificationAnimaliaAplousobranchiaDidemnidae

Herdman, 1886

Fig. 2A

Diplosomoides molle Herdman, 1886
Leptoclinum molle (Herdman, 1886)
Lissoclinum molle (Herdman, 1886)
Didemnum sycon Michaelsen, 1920

#### Specimen.

MZB. Asc. 00001

#### Habitat.

Coral limestone at reef crest.

#### Remarks.

Colonies were dome-shaped. Several morphotypes in colony shape and color exist in this species (i.e., brown, gray, white, large, and small type). These morphotypes can also be distinguished by the partial sequence of the cytochrome oxidase subunit I (COI) gene (M. Hirose et al. 2009; Hirose et al. 2010a). Brown-type colonies were found in the present survey. *Prochloron* cells were distributed within the common cloacal cavity. Testis and/or oocyte were found in some zooids.

**Figure 2.**
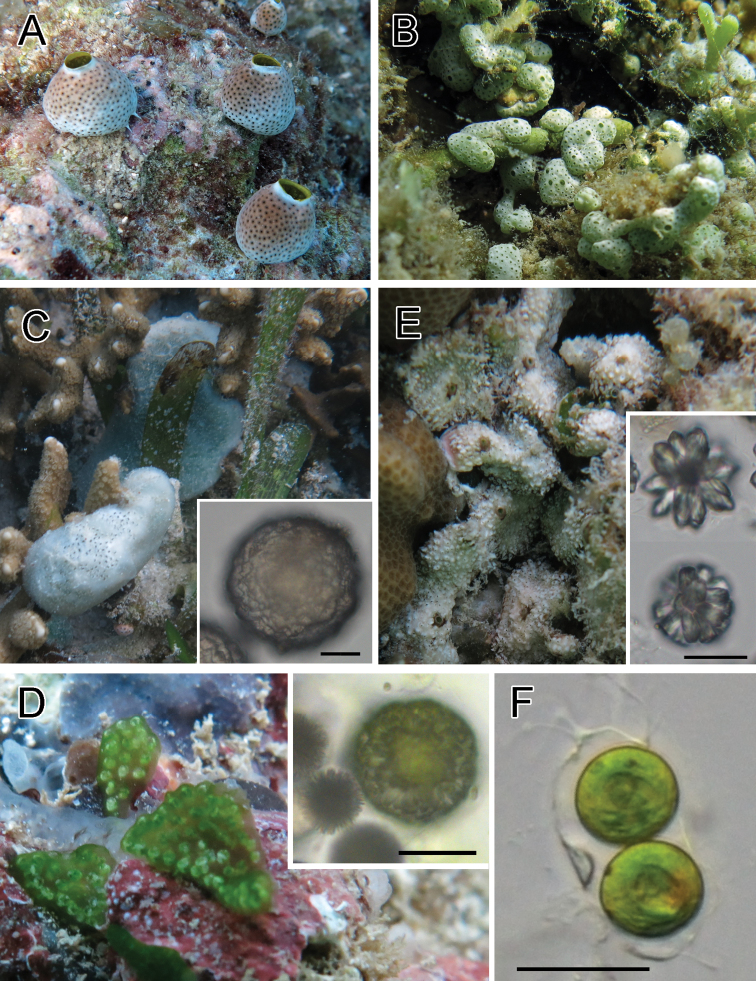
Photosymbiotic ascidians with tunic spicules. Colonies *in situ* and tunic spicules (inset) of *Didemnum molle* (**A**), *Trididemnum miniatum* (**B**), *Lissoclinum patella* (**C**), *Lissoclinum punctatum* (**D**), and *Lissoclinum timorense* (**E**). Tunic cells contain *Prochloron* cells in the tunic of *Lissoclinum punctatum* (**F**). Scale bars = 20 µm.

### Trididemnum miniatum

Taxon classificationAnimaliaAplousobranchiaDidemnidae

Kott, 1977

Fig. 2B

#### Specimens.

MZB. Asc. 00002

#### Habitat.

Dead coral skeletons and macroalgae in shallow back reef.

#### Remarks.

Thin sheets of colonies were white in exposed habitat and pale green in shaded habitat, depending on the amount of calcareous spicules in the tunic. *Prochloron* cells were distributed within the tunic.

### Lissoclinum patella

Taxon classificationAnimaliaAplousobranchiaDidemnidae

(Gottschaldt, 1898)

Fig. 2C

Didemnoides patella Gottschaldt, 1898
Didemnoides sulcatum Gottschaldt, 1898
Didemnoides ternatanum Gottschaldt, 1898
Didemnum meandrium Sluiter, 1909
Didemnum patella (Gottschaldt, 1898)
Didemnum ternatanum (Gottschaldt, 1898)
Leptoclinum patella (Gottschaldt, 1898)
Lissoclinum patellum (Gottschaldt, 1898)

#### Specimens.

MZB. Asc. 00003

#### Habitat.

Dead coral skeletons in back reef.

#### Remarks.

Colonies were thick cushions attaining about 10 mm in thickness. Tunic contains both stellate and globular spicules (Fig. 1C, inset). *Prochloron* cells were distributed within the common cloacal cavity. Some zooids had testes. Because of the large size, this species has been thoroughly studied for its natural compounds (e.g., Schmidt et al. 2012).

### Lissoclinum punctatum

Taxon classificationAnimaliaAplousobranchiaDidemnidae

Kott, 1977

Fig. 2D, F

#### Specimens.

MZB. Asc. 00004

#### Habitat.

Shaded side of dead coral skeletons in reef flat.

#### Remarks.

Colonies were irregularly shaped sheets. Globular spicules (Fig. 1D, inset) form a capsule-like aggregation enveloping each zooid. *Prochloron* was distributed within the common cloacal cavities and tunic. As reported in Hirose et al. (1996), algal cells in the tunic were contained in the tunic cells of the host ascidian (Fig. 1F).

### Lissoclinum timorense

Taxon classificationAnimaliaAplousobranchiaDidemnidae

(Sluiter, 1909)

Fig. 2E

Didemnum timorensis Sluiter, 1909
Didemnum voeltzkowi Michaelsen, 1920
Lissoclinum timorensis (Sluiter, 1909)
Lissoclinum voeltzkowi (Michaelsen, 1920)

#### Specimens.

MZB. Asc. 00005

#### Habitat.

Dead coral skeletons and clefts between coral limestones in back reef and shallow reef flat.

#### Remarks.

Colonies had linguiform projections of the tunic around the colony periphery and sometimes on the colony surface. Tunic contains both stellate and globular spicules (Fig. 1E, inset). *Prochloron* cells were distributed within the common cloacal cavity.

Because the zooids of *Lissoclinum bistratum* and *Lissoclinum timorense* are very similar in morphology, Monniot and Monniot (2001) proposed that *Lissoclinum timorense* is a junior synonym of *Lissoclinum bistratum*. Typical colonies of the two species are easily distinguishable by the presence or absence of linguiform projections on the colony surfaces, although intermediate forms between the two exist. Kott (2001) discriminated the two species based on the presence or absence of stellate spicules. However, the two species defined by spicule type could not be discriminated by molecular phylogeny based on partial COI gene sequences (Hirose et al. 2010b). We did not find *Lissoclinum bistratum*-type colonies in the present survey, although they are common in reef crests of the Ryukyus.

### Diplosoma gumavirens

Taxon classificationAnimaliaAplousobranchiaDidemnidae

Hirose & Oka, 2009

Fig. 3A, B

#### Specimens.

MZB. Asc. 00006

#### Habitat.

Shaded side of dead coral branches in reef flat.

#### Remarks.

Colonies were oval cushions and entirely green due to *Prochloron* cells distributed within the common cloacal cavities. A blue ring of structural color encircled each branchial siphon. Retractor muscle emerged from halfway along esophageal neck of a zooid. On each of the right and left halves of the branchial sac, there were five stigmata in the first (top), second, and third stigmatal rows and four stigmata in the fourth row (bottom). Here, we describe the pattern of stigma number as 5–5–5–4. This record is the first of this species from outside of the Taiwan–Ryukyu area.

**Figure 3.**
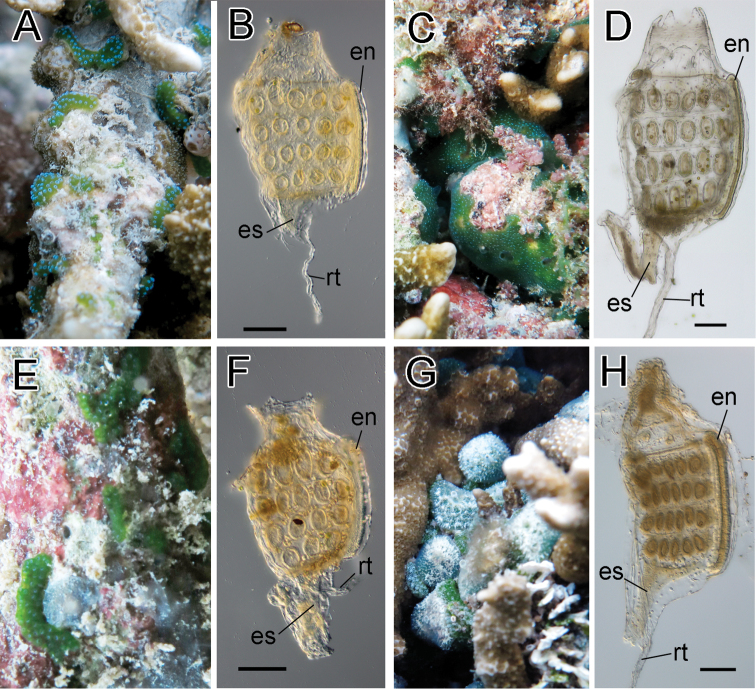
Photosymbiotic ascidians without tunic spicules (*Diplosoma* species). Colonies *in situ* and thorax of zooid of *Diplosoma gumavirens* (**A, B**), *Diplosoma simile* (**C, D**), *Diplosoma simileguwa* (**E, F**), and *Diplosoma virens* (**G, H**). **en** endostyle; **es** esophagus; **rt** retractor muscle. Scale bars = 0.1 mm.

### Diplosoma simile

Taxon classificationAnimaliaAplousobranchiaDidemnidae

(Sluiter, 1909)

Fig. 3C, D

Diplosoma midori (Tokioka, 1954)
Leptoclinum midori Tokioka, 1954
Leptoclinum simile Sluiter, 1909

#### Specimens.

MZB. Asc. 00007

#### Habitat.

Dead coral branch and coral limestone in reef flat and reef crest.

#### Remarks.

Colonies were irregularly shaped sheets and entirely green due to *Prochloron* cells distributed within the common cloacal cavities. Retractor muscle emerged from underside of thorax. The numbers of stigmata were 6–6–6–5. Some zooids had testes. Embryos were brooded in some colonies.

### Diplosoma simileguwa

Taxon classificationAnimaliaAplousobranchiaDidemnidae

Oka & Hirose, 2005

Fig. 3E, F

#### Specimens.

MZB. Asc. 00008

#### Habitat.

Shaded side of dead coral branches in reef flat

#### Remarks.

Colonies were irregularly shaped sheets and entirely green due to *Prochloron* cells distributed within common cloacal cavities. Retractor muscle emerged from underside of thorax. The numbers of stigmata were 4–5–4–3. This record is the first of this species from outside of the Taiwan–Ryukyu area.

### Diplosoma virens

Taxon classificationAnimaliaAplousobranchiaDidemnidae

(Hartmeyer, 1909)

Fig. 3G, H

Diplosoma viride Herdman, 1906
Leptoclinum calificiforme Sluiter, 1909
Leptoclinum varium Sluiter, 1909
Leptoclinum virens Hartmeyer, 1909

#### Specimens.

MZB. Asc. 00009

#### Habitat.

Basal parts on branching corals in back reef and reef flat.

#### Remarks.

Colonies were irregularly shaped sheets and entirely green due to *Prochloron* cells distributed within common cloacal cavities. Retractor muscle emerged from halfway along esophageal neck. The numbers of stigmata were 6–6–6–5. Some zooids had testes.

## Discussion

All photosymbiotic ascidians described here have also been recorded in the Ryukyu Archipelago, Japan (Hirose 2013 and references therein). Among the nine species, *Diplosoma simileguwa* and *Diplosoma gumavirens* were originally described from the Ryukyus in 2005 and 2009, respectively (Oka et al. 2005; E. Hirose et al. 2009b), and this report is the first to record these species outside of the Taiwan–Ryukyu area. The present records significantly expand our understanding of their range of distribution. The other seven species have also been described from the Great Barrier Reef (Kott 2001); thus, these species are widely distributed in the Indo–West Pacific. Among the five photosymbiotic ascidians recorded from Singapore, i.e., *Diplosoma simile*, *Lissoclinum bistratum*, *Lissoclinum punctatum*, *Lissoclinum timorense* and *Trididemnum cyclops*, (Su et al. 2013), two species, *Lissoclinum bistratum* and *Trididemnum cyclops* were not recorded in the present survey. These species are likely distributed in the Java Sea, considering that they are commonly found in West Pacific coral reefs. The recognition and identification of species are often important in field studies dealing with biocoenosis, and we hope that the present report will be helpful in future surveys and field courses in this area. Additional species, including undescribed species, are potentially distributed around Pari Island, considering its location within a biodiversity hot spot. Therefore, additional extensive surveys are necessary to characterize the photosymbiotic ascidian fauna in this area.

## Supplementary Material

XML Treatment for Didemnum molle

XML Treatment for Trididemnum miniatum

XML Treatment for Lissoclinum patella

XML Treatment for Lissoclinum punctatum

XML Treatment for Lissoclinum timorense

XML Treatment for Diplosoma gumavirens

XML Treatment for Diplosoma simile

XML Treatment for Diplosoma simileguwa

XML Treatment for Diplosoma virens
